# Exploring user experiences of a text message-delivered intervention among individuals on opioid use disorder treatment in Kenya: A qualitative study

**DOI:** 10.1371/journal.pdig.0000375

**Published:** 2023-11-06

**Authors:** Sarah Kanana Kiburi, Saeeda Paruk, Edith Kamaru Kwobah, Bonginkosi Chiliza

**Affiliations:** 1 Department of Medicine, Aga Khan University Hospital, Nairobi, Kenya; 2 Discipline of Psychiatry, University of KwaZulu Natal, Durban, South Africa; 3 Department of Mental Health, Moi Teaching and Referral Hospital, Eldoret, Kenya; Iran University of Medical Sciences, IRAN (ISLAMIC REPUBLIC OF)

## Abstract

Opioid use disorder causes significant burden of disease and treatment comprises pharmacotherapy and psychosocial treatment. Cognitive behavioral therapy is an effective psychosocial intervention used in substance use disorders treatment and can be delivered using digital approach. There is limited use of digital treatment among individuals with opioid use disorder in Kenya. This study aimed to describe the experiences and feedback from participants with opioid use disorder enrolled in a text-message intervention in Kenya. Qualitative data was collected from participants in the intervention arm of a feasibility trial testing a text-message intervention based on cognitive behavioral therapy. Data was collected using open-ended questions in a questionnaire and structured in-depth interviews amongst those who received the intervention. Framework method was applied for analysis. Twenty-four participants (83.3% males) were enrolled with a mean age of 32.5 years (SD9.5). Five themes were identified namely: (1) Gain of cognitive behavioral therapy skills which included: identification and change of substance use patterns; drug refusal skills; coping with craving and self-efficacy; (2) Therapeutic alliance which included: development of a bond and agreement on treatment goals; (3) Feedback on intervention components and delivery such as: frequency, and duration of the text message intervention; (4) Challenges experienced during the intervention such as: technical problems with phones; and barriers related to intervention delivery; (5) Recommendations for improvement of intervention in future implementations. The findings demonstrated participants’ satisfaction with intervention, gain of skills to change substance use patterns, highlighted challenges experienced and suggestions on improving the intervention among individuals with opioid use disorder. The feedback and recommendations provided by the participants can guide implementation of such interventions to allow acceptability, effectiveness and sustainability.

**Trial registration:** This study was part of a randomized feasibility trial. Clinical trial registration: Pan African Clinical Trial Registry: Registration number: PACTR202201736072847. Date of registration: 10^th^ January 2022

## Author summary

Opioid use disorder causes significant burden of disease among the substance use disorders. The recommended treatment comprises combination of pharmacotherapy and psychosocial treatment commonly cognitive behavioral therapy. Cognitive behavioral therapy (CBT) is a form of psychosocial intervention used in individuals with substance use disorders but there is limited use in low-and- middle income countries (LMIC) such as Kenya. Digital interventions through computers or phones have been used to deliver psychosocial treatment such as CBT and have shown effectiveness in improving outcomes among the individuals with opioid use disorder and other substance use disorders but use of these interventions is limited in Kenya and other LMICs. This paper reports the feedback and experiences of individuals who received CBT delivered via text-message. These findings show that the intervention was acceptable to participants who reported to have gained skills that helped them change substance use patterns. Participants also described challenges experienced and gave recommendations on how the intervention could be improved to benefit more people. The findings show that text-message can be used in psychosocial treatment delivery among individuals with opioid use disorder and can be used to guide implementation of similar interventions in similar settings within Kenya and other LMICs.

## Introduction

Opioid use is prevalent worldwide and among the substance use disorders (SUDs), it accounts for most of the adverse effects and mortality [1]. In Kenya, lifetime prevalence of opioid use among individuals aged 15–65 years in 2022 was 0.5% [2], and a systematic review on substance use research in Kenya, identified 21 studies with the prevalence of opioid use ranging from 1.1% among patients of Human Immunodeficiency Virus (HIV) treatment to 8.2% among psychiatric inpatients [3]. In addition, opioid use is associated with several negative effects [3].

The recommended treatment for opioid use disorders (OUD) include detoxification for management of withdrawal symptoms followed by long-term pharmacotherapy and psychosocial treatments [4,5]. Pharmacotherapy comprises use of medications for opioid use disorder (MOUD) which include methadone, buprenorphine and naltrexone [5,6]. MOUD are effective in improving outcomes among such as reducing substance use, improved retention in treatment, reducing infections and mortality and improving other outcomes such as quality of life [6,7]. Psychosocial treatments in OUD treatment include cognitive behavior therapy, community reinforcement approach, contingency management, counselling, motivational interviewing and acceptance and commitment therapy. These are used in combination with pharmacotherapy and help improve outcomes among individuals with OUD [5,6,8]. Psychosocial treatments have been offered in-person, but there is increase in delivery of these interventions via digital platforms with comparable effectiveness in SUD treatment including OUD [9,10].

Cognitive behavioral therapy (CBT), a structured and time-limited intervention, is used in the psychosocial treatment for variety of disorders using approaches that modify dysfunctional thinking and behaviour. In SUD treatment, this comprises analysis of thoughts, feelings and behaviors and then skill-training in order to achieve desired behavior with a focus on relapse prevention [11–13]. This entails training on change of drug use patterns through recognizing and then challenging problematic cognitions, identifying cues in the environment that may precipitate craving, developing skills to cope with craving, drug refusal skills training and improved decision making by identifying seemingly irrelevant decisions [13–15]. There is also development of self-efficacy whereby an individual feels equipped to change their substance use behaviour [13]. In OUD treatment, CBT has been used with improvement in substance use outcomes, retention in treatment and enhanced quality of life [16–18].

Digital platforms such as computers and mobile phones have been used in delivery of psychosocial treatment using the same theoretical basis as the in-person interventions. These interventions are referred to using various terms such as digital health, telehealth, telemedicine, mHealth (when delivered via mobile phones) and eHealth [9,19,20]. Among the digital platforms, mobile phones are the most commonly available especially in low- and middle-income countries (LMICs) [21,22]. Digital interventions offer more privacy, convenience, reach and are cost effective [23,24], whereby combining digital interventions and in-person therapy has better outcomes and may mitigate the challenges associated with either treatment approach and reduce the overall cost of treatment [25,26]. In addition, text-message based approaches are more accessible, since they require no internet connections and can be accessed even with basic phones which are cheaper and readily available [27,28]. Text messages also, are easy to use, can be delivered to individuals anywhere and can allow the messages to be tailored to individuals’ need in terms of content, timing and frequency [27,29–31]. Cognitive behavioral therapy has been delivered via digital interventions such as web-based modules, use of computers, smartphone applications and text messages [12,32–35].

Therapeutic alliance is a component of psychosocial treatment which influences SUD treatment outcomes [36]. Therapeutic alliance comprises (a) development of bond (interpersonal attachment) between the client and therapist, (b) agreement on the tasks (processes in the treatment) and (c) agreement on therapeutic goals (objectives of the treatment) and includes factors such as acceptance, empathy, congruence, confidence and openness [37,38]. In digital interventions, these dimensions may differ with additional themes such as availability (how accessible the intervention is) and intractability (extent of personalization and feedback from the intervention) [37]

Text messages used in SUD treatment have showed effectiveness and high acceptability [27,28,39,40]. Although few studies report use of text-message interventions in OUD treatment, the findings show feasibility, acceptability and potential efficacy in improving opioid use patterns and other outcomes [34,35,41]. Previous studies report on participant feedback after use of the intervention with recommendations on how these interventions can be improved. These include suggestions on type of messages perceived as acceptable or unacceptable, need for personalization of messages and provision of additional support such as individual psychotherapy sessions or use of multimedia [34,42,43]. However, there is limited research on use of digital interventions for OUD in LMICs especially in Africa [22].

In Kenya, text-message interventions have been used for other conditions such as HIV, maternal health and immunization. Participants in these interventions report them as acceptable with high satisfaction [44–47]. In Kenya, medications for OUD are available however retention in treatment remains low [48] and there is limited research on psychosocial interventions among individuals on OUD treatment [3]. Majority of individuals on OUD treatment have access to mobile phones and reported high acceptability for a text-message intervention [49]. To address this knowledge gap, a feasibility trial was carried out to assess the preliminary efficacy, feasibility and acceptability of text-message delivery of psychosocial treatment provided as an add on to methadone treatment. Following weekly text-messages for six weeks, there was a higher reduction in opioid use among participants in the intervention group compared those in control group although the difference was not statistically significant. Retention in methadone treatment was 93.3% at six weeks and 83.3% at 3 months follow up with high acceptability and satisfaction with the intervention based on quantitative assessment [50]. This paper aims to describe experiences and feedback from individuals who received the text-message intervention. This will give insight to the factors that need to be considered in implementation of digital interventions for SUD treatment in a larger sample.

## Methods

### Study design

This was a qualitative study conducted and reported using the Consolidated Criteria for Reporting Qualitative Research (COREQ) guidelines [51].

### Research team

#### Personal characteristics

The research team members are all psychiatrists at different levels of experience and practice. The lead author (SKK) was a PhD student at the time the study and was based at the methadone clinic where the study was conducted. SKK has training and prior experience in qualitative research.

#### Relationship with participants

The lead author, a female psychiatrist, had an established relationship with the participants as she was providing clinical services at the clinic at the time of the study. Participants were aware that the study was part of a PhD research that was assessing use of mobile phones in providing psychosocial treatment for individuals with OUD. This information was provided to study participants prior to enrolling in the study as part of the informed consent document.

### How trustworthiness of the data was established

There are four concepts that describe trustworthiness of data in qualitative research namely: credibility (confidence in the truth of the reported findings); transferability (degree to which the results can be transferred to other settings); dependability (stability of result over time); and confirmability (degree to which the results can be confirmed by other researchers) [52,53]. In this study these factors were ensured as follows:

Credibility: the data was transcribed word for word and the transcribed data was shared with all the authors for peer debriefing. The authors include verbatim responses from participants to support the reported results.

Transferability: the authors provide detailed description of the study methods and context and share the questions in the semi-structured interview guide to allow readers to understand what was done in the study. In addition, purposive sampling was done to ensure inclusion of participants in different categories as described below.

Dependability: the authors kept an audit trail by documenting all aspects of how data was collected and analyzed. There was continuous discussion throughout the analysis process and all authors agreed on the published results.

Confirmability: All documents used in data collection including the raw data in audiotapes (transcribed word for word) were kept and cross-checked throughout the analysis and report writing. The lead author kept a reflexibility journal after each interview and all authors ensured that the interpretation of data was grounded on the raw data by continuously assessing their biases and linking the results to the data. A thorough description of the data collection and analysis has been provided to allow readers to understand the processes in the study.

### Study site and participants

This study was conducted at a methadone clinic in Nairobi, Kenya, which is among eight public methadone clinics in Kenya. The clinic was started in 2017, and at the time of study, one thousand individuals were enrolled. Participants were eligible to participate if they were 18 years and older, had a diagnosis of OUD based on DSM5 criteria, were on methadone treatment, had current opioid use based on positive urine drug screen, owned a mobile phone with text-message capability, could read and send message in either English or Kiswahili and were willing to provide consent for the study. Recruitment occurred at the clinic by inviting the eligible individuals to participate. Those who did not provide informed consent were excluded.

### Intervention

The text message intervention in this study was based on CBT. The content of the messages in the intervention was adapted from the CBT manual [14]. The intervention comprised weekly text-messages for six weeks whereby for each week a different topic was discussed. Topics covered in the intervention and sample messages sent are summarized in Table 1. The text messages were in three parts: first to introduce the weekly module; second, teaching on the behaviour strategy; and finally, practical exercise for the strategies learnt in the module (homework). Participants were required to send the homework response to the therapist via text message and were allowed to ask any questions regarding the weekly module. The weekly text messages were sent same day to all participants. The feasibility trial was a 2-arm trial with 2:1 allocation with 30 participants in intervention arm and 16 in the control arm (standard treatment). Participants in the intervention were provided with weekly airtime worth Ksh. 50 ($0.5) as reimbursement for airtime used to provide responses. The intervention was conducted as an add on to standard treatment at the clinic. More details of intervention are available elsewhere [50].

**Table 1:** Summary of intervention modules and text-message content.

Week	Module	Objective	Content	Sample message sent
1	Functional analysis	Establish treatment goals and explain the psychosocial skills to be gained	Identify the motivation to change Identify triggers to using substances Identify the client’s strengths and treatment goals	Sometimes we encounter some situations in our daily life that influence use of drugs. These are called triggers…. Identifying what influences you to use opioids or other drugs is important so that you can know how to deal with the situation.
2	Coping with craving	To develop skills to recognize and cope with cravings for substance use	Identify high risk situations and develop a personal coping plan	Craving is when you experiences a strong desire to use drugs. Craving are associated with resuming or increasing heroin or drug use……. Ways to cope with craving include distracting yourself with activities such as walking or games…….
3	Shoring up motivation and commitment to stop	Clarify and prioritize treatment goals and address any ambivalence	Assess current readiness for change and ambivalence Revisit and clarify targets/goals Identify and cope with thoughts about substance use	This week we are going to revisit your treatment goals. This helps to see if we are on tract. Take a moment to consider how confident you are to stop using substances. Also we learn ways to cope with thoughts of heroin and other drugs. When you experience thoughts about using heroin or other drugs, here are some ways to deal with them: recognise the thought…..avoid the thought where possible.
4	Drug refusal	To develop drug refusal skills	Learning drug refusal skills and strategies to avoid contacts with people who use substances Application of assertiveness skills	It is likely that you will encounter situations where friends or other people offer you heroin or other drugs. Knowing how to react in this situation is helpful… First you need to avoid such situations where possible. If not possible to avoid, you need to have a specific strategy before you enter the situation.
5	Decision making skills	To identify and change thoughts commonly associated with substance use and develop decision making skills	How to identify seemingly irrelevant decisions that put one closer to using substances How to make decisions when confronted with a high-risk situation	During recovery one is likely to experience situations that may not seem to contribute directly to substance use, but move you closer to a high risk situation. When making a decision you can follow these steps. First, consider all the options you have; then think about all the consequences, both positive and negative, for each of the options; and select one of the options.
6	Problem-solving skills	To develop and apply problem-solving strategies Develop a support plan to address the problem	Identify the common problems Consider various approaches that do not involve substance use Termination of therapy	Everyone has problems from time to time. Most problems can be handled… Some problems may have arisen from drug use while other problems may contribute to drug use… To solve a problem, you need to use several methods until you get one that works for you.

### Study procedures and data collection

Data was collected from participants in the intervention arm in two parts. First, the post-intervention questionnaire had three open ended questions for all participants which were: (a) what did you like about the intervention? (b) what did you not like? (c) what can be changed to make the program better? The semi-structured interview had question such as “what was your overall experience with the program?; did you experience any challenge?”. These were followed by probing questions. Secondly, using purposive sampling, participants were invited to partake in individual semi-structured interviews until saturation was achieved. This was done face-to-face at the end of the text message treatment. The purposive sampling attempted to include individuals with different levels of participation in the intervention: Those who (A) did not respond to any text message, (B) responded to some messages, and (C) responded to all weekly messages. The interviews were carried out following the completion of the post-intervention quantitative questionnaire. These interviews were conducted privately during daily clinic visits by the lead author (SKK). No other participant was present in the room during the interview.

The interviews, which were audio-recorded, began with a brief introduction and participants were then asked to give their general experience with the intervention program and feedback on delivery of the intervention. The interviews lasted 10–20 minutes. Of the 30 participants in the intervention group, 24 responded to the open-ended questions (two were lost to follow up and did not fill the questionnaire and four left the questions blank). For the semi-structured interviews, saturation was achieved after eight interviews (these were part of the 24 who responded to open-ended questions) when no new data or information was being gathered from subsequent interviews. Those who did not respond to the questions did not provide the reason for non-response. The interviews were done in English or Kiswahili based on the preference of the participant. The questions used in the semi-structured guide are attached as S1 Text. No repeat interviews were carried out and transcripts were not returned to participants for comments or feedback.

### Data analysis

Data analysis was done via the framework method as described by Gale and colleagues [54]. The steps used in the analysis were as follows:

- Transcription: Data from the semi-structured interviews was transcribed word for word and data from the open-ended questions was typed into one document. Translation to English for the responses given in Kiswahili was done during transcription by SKK. These were combined into one document and uploaded to Nvivo software for coding
- Familiarization with data was done by listening to the interviews and reading the transcripts several times to identify potential codes.
- Generating initial codes was done by reading the transcripts line by line. Coding which was done using Nvivo software, was both deductive (based on the questions asked) and inductive (based on new topics emerging from the data).
- Developing and applying the analytical framework: after initial coding of two transcripts, common codes were identified and used to develop an analytical framework which involved grouping of codes to categories. This framework was applied in the remaining transcripts.
- Identifying and defining themes was then done by summarizing the connections between categories which comprised sub-themes. These were then summarized in a matrix with reference to relevant participant quotations.
- Interpreting the data and writing the report. The results were summarized in a narrative and participant quotations were included.

**Fig 1:**
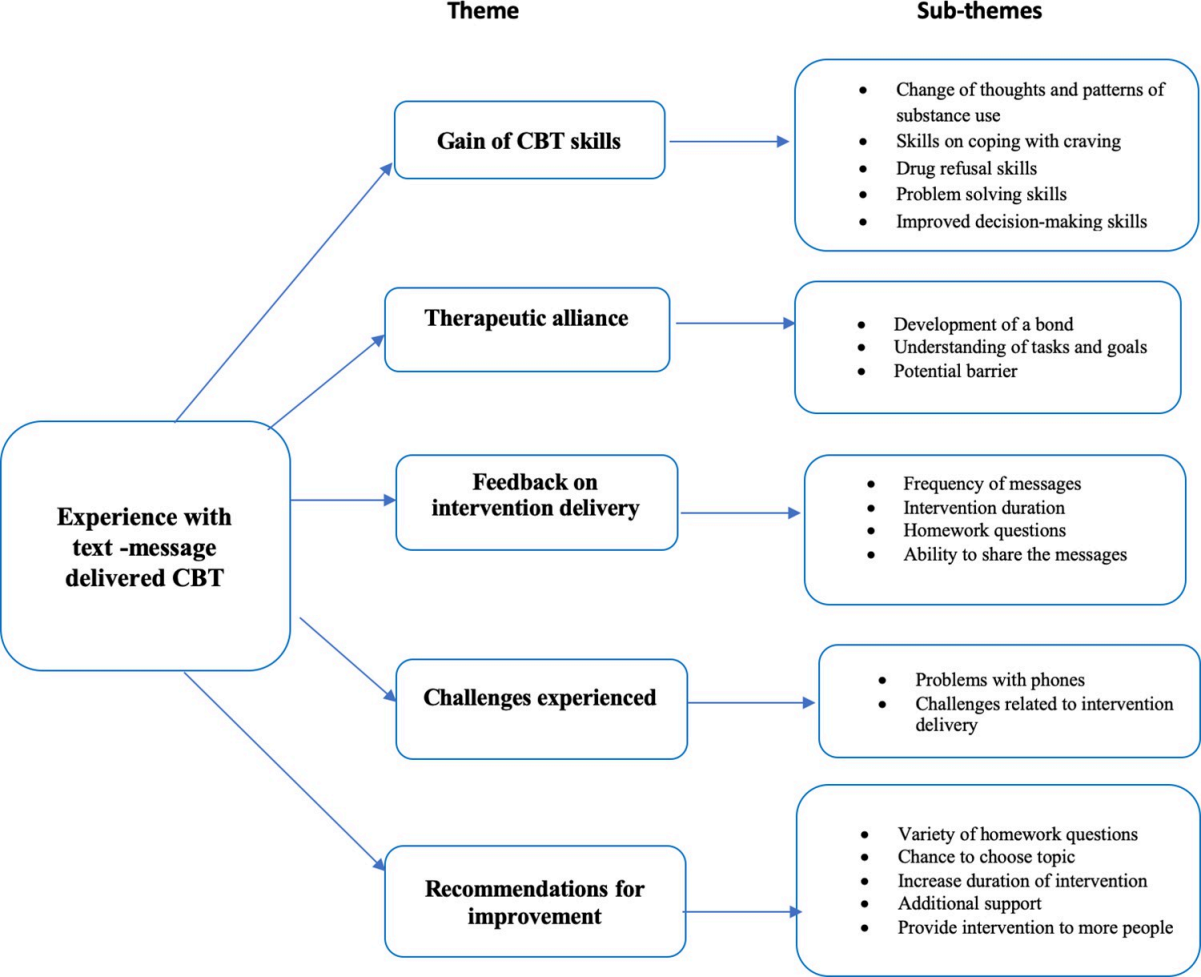
Summary of themes and subthemes.

A summary of themes and sub-themes is shown in Fig 1. Data coding and initial thematic framework was done by one author (SKK) and shared with the other authors (SP, EK, BC) for feedback, discussion and consensus. All authors were involved in the identification and defining of themes and agreed on all the themes included.

### Ethical approval and informed consent

This study was performed in accordance to the declaration of Helsinki. Ethical approval was granted the Biomedical Research Ethics Committee of the University of KwaZulu-Natal (UKZN) and Kenyatta National Hospital/University of Nairobi ethics review committee. All participants provided written informed consent prior to participation in this study.

## Results

### Sociodemographic profile of participants

The participants comprised mainly males (83.3%) and mean age of 32.5 years (9.5SD). Of note this is similar to pattern observed in the overall sample [50]. Table 2 presents a summary of the sociodemographic and clinical profile for the study participants.

**Table 2:** Summary of sociodemographic and clinical profiles of participants.

Variable	Category	Frequency (N = 24)	Percentage
Gender	Male	20	83.3
	Female	4	16.7
Age	Mean (SD); Median; Range	32.5(9.5); 29.5; 23–63	
Education Level	Primary and below	13	54.2
	Secondary/ High School	5	20.8
	College / University	6	25.0
Employment status	Employed	14	58.3
	Unemployed	10	41.7
Marital Status	Married	8	33.3
	Divorced or separated	9	37.5
	Single	7	29.2
Age at first substance use	11–15 Years	5	20.8
	16–20 Years	16	66.7
	21+ Years	3	12.5
Ever used substance by injection	No	12	50.0
	Yes	12	50.0
Treatment duration	1–3 Years	14	58.3
	4–5 Years	10	41.7
Status at baseline	Continuing treatment	11	45.8
	New	5	20.8
	Reinduction	8	33.3

### Themes

Five themes were identified namely: Gain of CBT skills; therapeutic alliance; feedback on intervention components and delivery; challenges experienced during the intervention and; recommendations for improvement. Several subthemes were identified for each theme.

### Themes

#### 1. Gain of CBT skills

This theme discusses how participants reported to have gained CBT skills from the intervention. This helped them improve substance use behaviour and other areas in their daily lives. Several subthemes were identified as follows:

**Understanding and changing thoughts and patterns of substance use:** The participants reported getting information about substance use and the effects of substances. This was based on psychoeducation which was a component of the intervention. This facilitated their understanding on substance use and were able to change their patterns of substance use.

“*It helped me a lot to shape my problem in my drug addiction*. *To know the bad part about it and how I should avoid drugs…*. *The advice that I got is really helpful to me and has helped my life*. *It gave me advice I have not received from anywhere else*.*”* (Male, 37 years)

“*The most basic and helpful thing I learnt is how to cope with drug use*. *In case you are in a delicate situation and there are drugs in your surrounding how to stop your thoughts and not use*.*”* (Male, 42 years)

Participants reported that they had gained information on triggers and high-risk situations. Having the ability to identify the triggers such as friends and negative emotions, helped them avoid using substances. This was attributed to the information provided through the text messages and perception that the program kept them busy hence were able to avoid using substances.

“*Most of the messages were about triggers*. *For triggers you know*, *it is mostly friends*, *the messages gave advise to avoid such friends who are using drugs*.*”* (Male, 37 years)

“*Actually*, *the program was good because the things we discussed about substance use gave advice and motivation to stop using drugs*. *It was good and it helped to keep me busy…*. *For example*, *there was a time my things were not going well and I was feeling stressed*. *Previously this is what made me use drugs but this program kept me busy and made me to feel less stressed about my problems*.*”* (Male, 51 years)

**Coping with craving:** Participants reported to have gained information about craving and developed skills to deal with the craving which consequently resulted in reduced substance use. Also, the flexibility of the intervention whereby information on how to deal with cravings was available during the time of experiencing it without having to come to the clinic was reported as beneficial.

“*The program was fantastic*. *One because of the concept of time*, *it takes place at different times*, *you can read the text in the morning or evening*. *You don’t have to be here to see a counsellor*. *Maybe you could be going through a challenge*, *let’s say craving*, *and then you get a message about craving or maybe it could be withdrawal*, *then you are able to deal with the issue at hand during that time*. *I think it is a good program*. *I prefer it that way*.*”* (Male, 27 years)

**Drug refusal skills:** Participants discussed gaining skills on how to refuse drug offers, which were mainly from friends. They were able to identify the friends who offered substances to them and by keeping away from these friends, it was easy for the participants to stay without using substances.

“*For me I would say that the text-message program helped me a lot because you gave advise on how to avoid friends because they are the ones that lead me to use substances*. *I got help on what to do to stay away from those friends*.*”* (Female, 33 years)

**Problem-solving skills:** In addition to reduction or stopping substance use, participants reported that the intervention had benefits in other areas of their life such as being able to plan daily life, developing a financial plan, anger management, dealing with peer influence and improved relationships.

“*I found the program to help me in areas like planning my finances and how to approach my day to day routines…*..*started selling things like earrings slowly it picked up… Some of those things we are not taught here at the methadone program*, *how you can have financial planning and growth*.*”* (Male, 34 years)

“*I learnt about anger management*, *overcoming peer pressure*. *I really enjoyed the program of which it has created a positive impact to me and also to my husband*.*”* (Female, 29 years)

**Improved decision- making skills:** Participants found they gained information on improving decision making to avoid using drugs based on the information shared in the text messages.

“*Regarding substance use*, *I learnt very many good things*. *The messages gave morale and a sense of direction*. *There was advise on things to do to forget about substance use*. *For example*, *it would say this will not help but this will help*. *I found that useful*.*”* (Male, 42 years)

**Self-efficacy:** Participants expressed self-efficacy and confidence to be able to stay away from substances. They felt that they were well equipped to continue with their change in substance use behaviour even after the intervention period. Participants also observed that the effect of the intervention also depended on their motivation and effort of the individual in applying the information provided.

“*Thank you for the advice*. *I am positive I can stop using drugs with the support given*.*”* (Female, 23 years)

“*The program was helpful in advising someone on how to stop using heroin*. *When you were asked the questions*, *and you responded and followed the advice*, *it was helpful*, *but if you don’t follow the advice then you don’t benefit*.*”* (Male, 36 years)

#### 2. Therapeutic alliance

Participants expressed developing a therapeutic alliance during the intervention. First there were comments that showed a bond between the therapist and the participants who felt that the feedback provided enhanced the interactive nature of the intervention which is important for a therapeutic alliance. Although not able to interact physically with the person sending the messages, participants felt connected with the therapist.

“*The messages were good*. *The person sending was also kind*, *used good language and treated us well*. *It was easy to understand…*. *the approach and confidentiality was good*.*”* (Male, 37 years)

“*I liked the fact that one could get counselling services at any time of the day without having to appear physically at the clinic…*. *the intervention was diverse*, *it touched on different topics*, *it was well explained and you could get feedback*.*”* (Male, 27 years)

Another aspect of therapeutic alliance is agreement on tasks and goals. Participants expressed to have understood the expectations during the intervention and were able to follow through the tasks as expected.

“*Because it was like a class or lecture*, *first you got two messages explaining and then a question for homework*. *This helped me to think around the topic and question*. *The language used was also positive*, *English was easier for me*.*”* (Male, 51 years)

“*The information in the messages was great*. *It encouraged you to stick to your goals what you decided to do or what you have been doing*. *For example*, *you don’t go back to your past*, *what you have been doing*. *For*, *example if tomorrow you get angry about something*, *you do not go to use drugs like in the past*.*”* (Male, 25 years)

The element of flexibility and being allowed time to respond also enhanced the therapeutic alliance as the participants did not feel rushed hence could work on the homework at their own pace. Having a chance to choose the language they were more comfortable with further enhanced the alliance.

“*The program was good because the things we discussed about substance use*, *it gave advise and motivation to stop using drugs*. *Even when you did not respond to the questions immediately*, *you were given time to respond later*.*”* (Male, 51 years)

“*I liked getting information on how to reduce using substances and being given an option to choose between English and Kiswahili languages*.*”* (Male, 25 years)

A challenge that was reported as a possible barrier to optimal therapeutic alliance was the lack of face to face sessions with the therapist. Participants felt that including some level of in-person interaction with the therapist in addition to the messages, would improve the intervention.

*“Message alone is not enough*. *There is need for someone to have a session with counsellor for more detailed discussion*. *You see*, *the message can give advice*, *but it may be difficult to do follow the instructions in the message*. *But when someone meets with counsellor*, *the counsellor can help to guide more in order to follow the advice given in messages more easily*.*”* (Male, 37 years)

#### 3. Feedback on intervention component and delivery

This theme discusses the perception of participants on the delivery of the intervention such as the duration and frequency of the messages.

**Frequency of message and intervention duration:** There were mixed feelings about the frequency of the messages. Some participants were content with the frequency of the messages while others felt that the messages needed to be more frequent.

*“For me I think the frequency of messages- getting the messages once a week- was not enough*. *I would prefer a minimum of 2–3 times in a week*, *like Monday*, *Wednesday and Friday*.*”* (Male, 34 years)

On the other hand, one participant reported that the six weeks duration was long and suggested shorter duration such as four weeks.

*“However*, *something I would add*, *is that instead of the 6 weeks I would say maybe to go for 4 weeks because 6 weeks is too long*.*”* (Male, 34 years)

**Homework questions:** Participants were receptive of the homework questions and reported them as appropriate, easy to follow and a tool that helped them to understand the information better.

*“The questions were not bad by the way*. *The questions helped me to evaluate myself*. *It gave an idea on the things that I can do or the things to avoid*, *to get myself from using drugs*. *I would say it really helped*.*”* (Male, 37 years).

**Ability to share information with others:** Participants reported that they shared messages from the intervention with their friends to influence their substance use behaviour.

*“The homework questions were good*. *I liked it and it helped me*. *It also helped my friends because since I would not want them to continue the way they were*, *I shared with them the messages and advice from the program*.*”* (Male, 34 years)

#### 4. Challenges experienced

In this theme participants identified challenges related with the intervention which were related to phone damage or loss and some components of intervention delivery.

**Problems with phone:** Some participants reported problems with their phones which prevented them from completing all the homework questions or made them unable to go back to read the messages to remind themselves on what they had learned. Participants indicated that having a chance to come to the clinic for an in-person session would help during the times when they experienced challenges with the phones.

*“I have changed my phone recently*. *This is a new phone*. *Therefore*, *I did not answer all the homework questions*. *I may have missed 1 or 2 when I changed phones*. *Sometimes if you missed or delayed responding to one*, *you find another message came requiring you to respond so you end up not responding to all homework questions… since the screen broke I wasn’t able to go back to the messages*.*”* (Male, 25 years)

*“Sometimes I had a challenge with my phone being off*. *In that case*, *having an option to come to the clinic would be good*. *Maybe to discuss as a group*.*”* (Male, 42 years)

For some participants the challenge was having airtime to respond to the questions. Despite them being provided with weekly airtime reimbursement during the study period, some said that the airtime got spent in other ways.

*“The challenge was the airtime was not enough and was being used up by (the telephone company) due to somethings I may have subscribed to unknowingly hence I ended up using my money*.*”* (Male, 34 years)

Frequent phone loss was expressed as a challenge that can hinder one from participating. One participant was glad that he did not lose his phone during the intervention period. There are those who missed some weekly messages due to problems with phone.

*“Most times I lose my phone and would not have benefited but it was good during this time of program I had a phone*.*”* (Male, 51 years)

**Challenges with delivery of intervention:** Some participants reported challenges with understanding some message based on the structure, with certain messages being described as long necessitating need to get help so as to comprehend.

*“In terms of length*, *I would suggest using short messages that are easy to understand*. *There are a few times*, *I found some messages to be hard*, *and I would go look for someone*, *with more education to explain to me…*. *I answered all the questions but I would prefer if the message was shorter and easier to understand*.*” (*Male, 36 years)

*“The content was okay although sometimes it was very long and tiresome to read*. *Especially if the message is sent in the evening when the mind is tired*. *I prefer they send the message in the morning for those long messages*.*”* (Male, 27 years)

Another concern expressed by participants was getting the same message more than once which occurred occasionally. This was reported to make the intervention boring.

*“Sometimes you got the same message within even more than two times this makes you get bored*.*”* (Male, 26 years)

#### 5. Recommendations for improvement

This theme discusses the various suggestions from participants on how they felt the intervention can be changed to be more useful and benefit more individuals at the clinic.

**Include a variety of homework questions and chance to choose topic:** Participant suggested that to improve on intervention delivery, there was need to make the content easy and provide options of homework questions. This would give a person an opportunity to choose a question that they understand and feel equipped to respond well.

*“The two messages and homework were okay but for homework questions I would prefer it was made easier*. *For example*, *have questions ‘a’ and ‘b’ with options for someone to choose the one that is easier to them*. *This would help even those with low education to respond*. *There was a day I was with a friend*, *I shared the question with him and told him to answer but it was a challenge for him*.*”* (Male, 51 years)

Participants also suggested allowing them to choose the topic of discussion so that the person and the therapist can focus in area that the individuals felt they needed more support.

*“I think it is better to have the messages sent at random*. *But then it is better to tell someone to say the topics they have challenges in*. *We were never given that chance to do that*. *For example*, *one may be asked if there something that they don’t like and then from there the counsellor goes through the list to address the issues*.*”* (Male, 27 years)

In addition, participants expressed need for more content to be included in the program such as more information on methadone treatment. This would enhance their knowledge on substance use disorders treatment which can help improve other treatment outcomes such as retention in treatment.

*“For example*, *in addiction*, *you know even methadone treatment is about addiction*, *the message can be used to help people understand how methadone works*.*”* (Male, 42 years)

**Increase duration and/ frequency of message:** Participants suggested increase in frequency of message delivery, with message delivery several times a week instead of once. They also felt a longer duration of intervention would help in improving the treatment outcomes over time and that more frequent messages would allow them to discuss more topics and enable the intervention to be delivered over shorter period.

*“I feel it is better if the message was given more days*, *maybe daily Monday to Friday so that every day you discuss something different*.*”* (Male, 36 years)

*“If the messages are given daily*, *Monday to Friday*, *3 weeks to one month would be enough instead of the six weeks*.*”* (Male, 37 years)

Participants noted that the longer duration would benefit those who may need more time to recover.

*“To increase the time of the intervention to be more efficient to those who need more time or take time to heal*.*”* (Male, 30 years)

**Provide additional support:** For some participants the intervention was sufficient as delivered. However, some participants gave suggestion on adding elements to improve the intervention. This included addition of phone calls and face-to face visit with the psychologist for individual and group therapy

- Phone calls: both audio calls and video calls were cited as beneficial if included in the intervention.

*“Messages were okay*. *But maybe for those with smartphones we can do video calls or something like that*.*”* (Male, 27 years)

- Face to face visit: Participants recommended visits to the clinic either for individual sessions with the counsellor or group therapy. They felt that the visit with the counsellor would help clarify any difficulty experienced and provide a platform for what should be included in subsequent messages.

*“I feel that the messages alone are okay*. *However*, *having a chance to see a counsellor would be better*. *For example*, *since the messages are once a week*, *it can be planned that after receiving the message*, *someone then gets a session with the counsellor*. *Then the counselor would review the topic with you and see if your understanding is okay or not*.*”* (Male, 42 years)

Participants suggested including group sessions as part of the intervention. This included virtual meetings with an option of meeting physically at the clinic.

*“I can advise we should form a group therapy in which a client come to share with their counselor about most difficult time in recovery and also introduce one-on-one session with the client and friendship with like-minded people who are trying to recover*. *How to guide the client in the journey of recovery*.*”* (Male, 42 years)

- Include family members: Participants cited the role of including family or an individual’s support system in the intervention so that they can be aware of what is happening. They further mentioned that this could help in improving accountability to the patient even if the family member does not actively participate in the discussion.

*“Sometimes it would be good to communicate with someone’s family and tell them this and this is happening*. *It can even be conference call*, *maybe with a parent*, *client and counsellor*. *The family therapy can also be a WhatsApp chat*, *so that the message I get*, *my mother or someone else also gets them*. *The other person may not respond but at least they are able to see the conversations that are going on in the chat*. *That is something I would like to be added*, *a group chat with the family*.*”* (Male, 27 years)

### Offer the intervention to more people at the clinic

Most participants said that they would recommend the intervention to others with suggestions of including the intervention as part of routine services to everyone at the clinic.

*“All I can say is that I would like you to help more people…*. *I recommend it to be included as an option in the psychosocial department especially for those clients living far from the clinic*.*”* (Female, 29 years)

Participants further gave suggestion in how the program could benefit more people, including those with no phones by using written material as an alternative or by phone calls.

*“The program should be offered to everyone especially those with phones*. *For those without phones the same information can be typed or printed on paper and it can have an impact*. *If being given as a paper the strategy that can be used*, *is when people are taking their medication at the pharmacy*.*”* (Male, 27 years)

*“Maybe you help those who can’t read so that instead of SMS you just call them*. *To me that is what was in my mind*.*”* (Male, 25 years)

Participants felt that if the intervention was offered to everyone at the clinic, many would be willing to participate. However, this would depend on the motivation of the person and their attitude towards the intervention.

*“For those people who are willing*, *the intervention would really help their life in a great way*. *Because most people are not stable but keep relapsing to heroin use and then coming back*. *But if they are given the messages to read*, *and one settles down to read and think about what the messages say*, *they can learn a lot and stop using heroin and other drugs and also think about what they want in life … I would say it depends with someone’s motivation*. *If someone considers it to be beneficial they would enroll*, *if they think it is not helpful*, *then they would not*.*”* (Male, 42 years)

## Discussion

In this study, participants with OUD provided feedback on their experience with a text-message CBT intervention. Five main themes were identified namely: Gain of CBT skills; therapeutic alliance; feedback on intervention components and delivery; challenges experienced during the intervention; and recommendations for improvement. These findings provide insight on usefulness of the intervention in the study population. For example, while the participants reported perceived benefits from the text message intervention, they also provided feedback on the challenges experienced and suggestions on how the intervention could be modified to enhance the benefits.

The first theme was gain of CBT skills based on the relapse prevention model. This has been seen following face-to-face CBT interventions with this gain of skills being seen as one of the mechanism that influences treatment outcomes [55]. Similar findings have been shown among participants using digital interventions including text-message intervention [30,34,41]. This shows role of digital interventions in improving cognitive skills that enable one to avoid substance use and need for further evaluation in future studies [33]. Participants reported to have developed self-efficacy following the intervention. This was also demonstrated using quantitative measure in the same sample where there was significant improvement of self-efficacy in the intervention group compared to the control group [50] and in another study using CBT approach among individuals with OUD [35]. This is significant since self-efficacy helps improve outcomes in individuals with SUD [56] and shows potential for text message intervention to influence substance use in this study population.

Participants reported developing a therapeutic alliance whereby they described establishment of bond and agreement on goals and tasks. This has been demonstrated in previous studies using digital intervention among individuals with depression and anxiety [38,57,58]. Participants also identified some barriers for therapeutic alliance including need for personalized messages and inclusion of in-person sessions. This gives insight to strategies to be applied in future studies to improve therapeutic alliance in digital SUD treatment. Among the strategies recommended for improving therapeutic alliance in digital interventions include: tailoring and personalization based on participant preference or characteristics identified during assessment; dialogue approach thorough feedback and reminders; providing social support and; using features that show the user that the system is credible [37]. These can be explored in future studies. In addition, future studies need to use objective measures of therapeutic alliance such as use of validated tools for more standard assessment and reporting [59,60].

Participants also gave feedback regarding the intervention in terms of frequency of messages, duration of treatment and treatment delivery. Although majority were satisfied with the features of the intervention, there was mixed findings in terms of duration and frequency of messages. This pattern has been reported in previous studies assessing use of text message among individuals with OUD [41,61]. A survey among the same study population prior to implementation of the intervention found similar reviews whereby 65.5% preferred one message per day, 39.7% were willing to receive messages once a week while 22.4% wanted daily messages [49]. This further shows role of personalization and tailoring of messages according to patient preference as suggested in previous studies [31,34,41]. Future studies need to consider tailoring the frequency and duration of messages based on individual preferences.

The fourth theme focused on challenges experienced with the intervention. First, there was problems with phone such as loss or technical issues. Also, despite being provided reimbursement for airtime during study period, participants still reported challenges with airtime. High phone turnover is a common problem among individuals with SUD [30,62] hence this is a challenge that needs to be considered in future studies and develop strategies to mitigate during implementation. These strategies may include: frequent inquiries by staff on status of phone ownership and asking participants to update their phone number details whenever they change phones during the study period [30,41]. In some studies, participants are provided with phones e.g. [63] but this may not be feasible in long-term care of patients if the intervention is to be provided as routine care.

Another challenge noted was based on how the messages were delivered such as lengthy messages and getting same message more than once. This has been reported in previous studies among individuals with OUD and participants give recommendation for having both automated and tailored text messages combined during the intervention period [34,41]. The perception on lengthy message and difficulty in understanding by participants may also be related to low education and cognitive impairments observed among individuals with OUD and/ other SUDs such as deficits in information processing, attention, memory and executive functioning [64–66]. Strategies on how to compensate for this cognitive deficit during behaviour interventions in these patients include having structure and consistency, simple and clear language, using a range of modalities, memory aids e.g. reminders and frequent assessments with immediate feedback during the intervention [64,67]. These point to potential barriers that need to be considered during future implementation of a text-based intervention in this study population.

Participants also gave recommendations on how to improve the intervention including providing it to more people at the clinic, improving delivery of the messages and adding more features. This is consistent with findings in a study by among individuals on buprenorphine treatment where 95% felt treatment should be offered to everyone at the clinic [68]. The suggestions also specified other ways in which those with no access to phones could be included such as by having them read materials through written pamphlets. This has been used in previous studies among individuals with OUD and has shown effectiveness in improved substance use with similar effect as a digital intervention [69] and can be explored in future studies. Another aspect was addition of other features such as a human contact with inclusion of individual and group therapy sessions with a therapist as reported in previous studies [41,42]. These suggestions also agree with strategies given to improve effectiveness of text message interventions such as using a behaviour change theory in design of the intervention (which was included in the current study), tailored and personalized messages [30,31,40], and adding multimedia function to increase interaction [34]. The recommendations provide a useful guide on factors that need to be considered and addressed during the implementation of the text message intervention to a larger population to enable sustainability.

### Strengths and limitations for this study

The strength of this study is based on being the first to report experiences of participants receiving a text message intervention for OUD treatment in Kenya. This fills the knowledge gap for digital intervention use among individuals with OUD in a LMIC setting and provides valuable insight to guide development and implementation of future text-message or other digital intervention.

Limitations to the study include the following: First, study was among individuals on OUD treatment at one clinic hence may not be generalizable to other patient populations. However, these finding can give insight in implementation of a similar intervention in other settings. Secondly, the interview gathered feedback from those who completed the intervention hence did not get feedback from the participants who were lost to follow up despite them being invited for the interview. This is because none of participants who were lost to follow up was available for the post-intervention assessments.

Third, the interviews lasted 10–20 minutes which was relatively short and may have missed in-depth information. Possible reasons for this include: (1) Time limit as the interviews were conducted during the daily visit to the clinic and participants needed to report back to work. This was partly because participants had spent additional time to complete the quantitative questionnaire assessment before the interviews. (2) The respondents had responded to the open-ended questions in the questionnaire and felt some of the questions asked during the interview as repetitive hence provided brief responses. Of note is that the interview duration excluded the preliminaries such as introduction of study as this had been done prior to the in-depth interviews. However, despite the short duration, participants were able to give insight to their experiences with the intervention. Similar short duration has been reported among patients with OUD in this patient population [70] and other setting [71].

Fourth, the initial coding was done by one author. Whilst the recommendation is to have at least two authors do the coding [54], this was not possible at the time of the study. However, the lead author shared all the transcribed interviews and the initial code book with all authors for feedback. All authors were involved in the identification and defining of themes.

Fifth, there is potential for biases such as social desirability whereby participants may have felt the need to give positive review of the intervention and/ conceal their substance use behaviour post-intervention as is common among individuals with SUD [72]. Therefore, the positive reviews need to be interpreted with caution.

## Conclusion

The findings in this study revealed an overall positive experience, satisfaction with the text message intervention and gain of skills to support change in substance use behaviour. In addition, participants described challenges experienced with the intervention and gave recommendations on how the intervention could be improved.

This provides valuable insight on application of text message intervention in delivering psychosocial treatment among individuals with OUD. Further studies are recommended to assess the implementation of the intervention to a larger population while incorporating the suggested modifications to test the effectiveness. In addition, these findings need to be correlated with the quantitative data findings from the same study that showed promising efficacy, feasibility and acceptability of the text-message intervention [50] to inform policy and future research.

## Supporting information

S1 COREQ Checklist Consolidated Criteria for Reporting Qualitative Research (COREQ) checklist. (PDF)

S1 Text Semi-structured interview guide. (DOCX)
