# Supplementary material for: Exploring user experiences of a text message-delivered intervention among individuals on opioid use disorder treatment in Kenya: A qualitative study
Source: PLOS Digit Health. 2023 Nov 6;2(11):e0000375. doi: 10.1371/journal.pdig.0000375 (PMC10627438; doi:10.1371/journal.pdig.0000375)
Supplement: S1 Text — (DOCX) [file pdig.0000375.s002.docx]

**Semi-structured interview guide**

1. What was your overall experience with the program?

- After enrolling did you feel satisfied with the service you got?
- Is there anything particular you liked most about the program?
- In what way did it help?
- Did you achieve your goal?
- Did you find the program to benefit in other areas of your life other than substance use?

1. What was your view about the message content?

- Did you respond to all the questions?
- Were messages only enough? what more can be added?

1. What are your thoughts about the homework questions?
2. What is your view about the once a week frequency of receiving messages

- What about six-week duration?
- Which would you prefer, the message to be sent random or to be allowed to choose the time to receive the message?

1. Did you experience any challenge? Please explain.

- Anything you did not like

1. Do you think the intervention would benefit more people if offered to everyone at the clinic?

- Would you recommend it to a friend?

1. Do you think that people would enroll if there was no airtime being offered?
2. If the program was to be delivered to more people, are there things that you feel should be improved to make it better?

- Anything more that can be added to improve
